# 1445. Public Health Containment Response Detects Possible Transmission of Echinocandin-Resistant *Candida auris* in a Long-term Acute Care Hospital

**DOI:** 10.1093/ofid/ofad500.1282

**Published:** 2023-11-27

**Authors:** Brenda M Brennan, Sara E McNamara, Ann M Valley, Diane Podzorski, Meghan Lyman, Lindsay Parnell

**Affiliations:** Michigan Department of Health and Human Services, Lansing, Michigan; State of Michigan, Lansing, Michigan; WISCONSIN STATE LAB OF HYGIENE, Madison, WI; Wisconsin State Laboratory of Hygiene, Madison, Wisconsin; Centers for Disease Control and Prevention, Atlanta, Georgia; Centers for Disease Control and Prevention, Atlanta, Georgia

## Abstract

**Background:**

*Candida auris* is an emerging fungal pathogen which is often multidrug-resistant, can cause severe infections, and spreads readily in healthcare settings. While echinocandin resistance (ech-R) remains low in the United States (< 5%), the number has been increasing, which is concerning because echinocandins are first-line therapy for *C. auris* infections. In May 2022, the first ech-R *C. auris* isolate in Michigan was detected at long-term acute care hospital (LTACH) A and enhanced lab surveillance was conducted to assess potential for ech-R transmission.

**Methods:**

Upon detection of *C. auris* in a urine specimen at LTACH A in March 2022, a containment response was initiated, which included clinical surveillance and colonization screening of patients at LTACH A. *C. auris* colonization screening testing and antifungal susceptibility testing (AFST) was performed at Wisconsin State Laboratory of Hygiene and whole genome sequencing (WGS) by the CDC. Ech-R was determined using tentative breakpoints defined by CDC.

**Results:**

From Mar 2022 – Sept 2022, 15 *C. auris*-positive specimens were detected from 12 patients at LTACH A (5 clinical specimens, 10 screening swabs). In May 2022, AFST on a blood isolate from a *C. auris*-colonized patient showed resistance to azole and echinocandin classes. This patient had no history of prior echinocandins use. AFST conducted on 12 available isolates (5 clinical, 7 screening) detected ech-R in 10 (83%) isolates (3 clinical, 7 screening); all isolates were resistant to azoles and susceptible to polyenes. WGS was performed on 4 isolates and revealed 3 closely-related clade III isolates (all ech-R), suggesting possible facility transmission, and 1 clade IV isolate (susceptible), suggesting a separate introduction.

**Figure:**
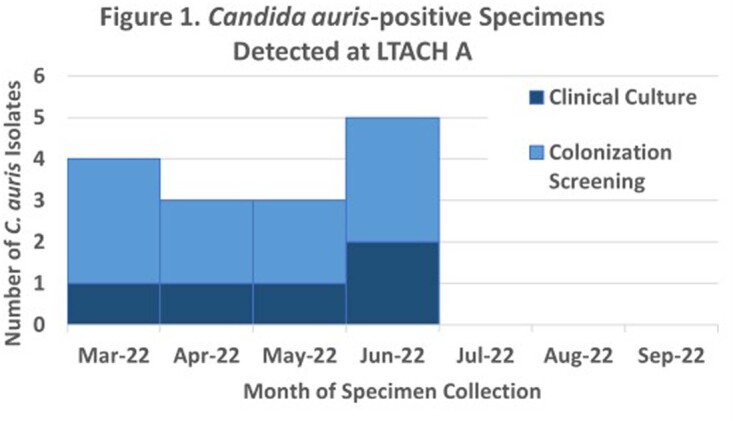

**Figure:**
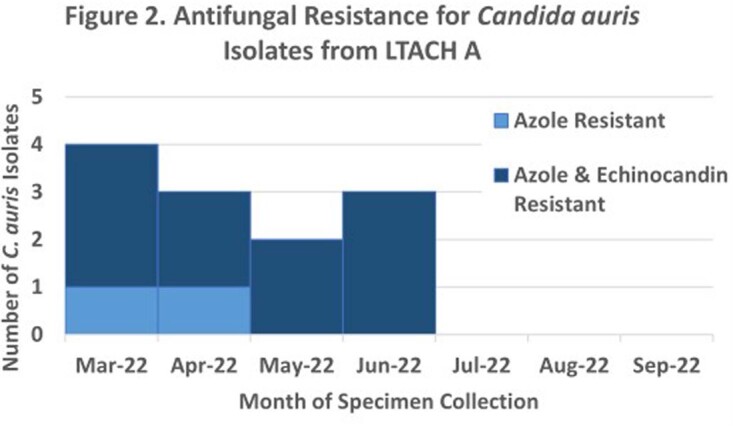

**Conclusion:**

Multiple ech-R *C. auris* isolates detected among patients overlapping in the same facility and with close genetic relatedness provide further evidence that echinocandin resistant strains can be transmitted among patients and raise concerns about spread to a larger population of patients without prior echinocandin use. Enhanced surveillance for *C. auris* detection and antifungal resistance, public health reporting, and robust infection prevention and control measures are critical to containing the spread of concerning resistance.

**Disclosures:**

**All Authors**: No reported disclosures

